# Improving Discharge Summary Follow-Up Documentation in a Major Trauma Center: A Two-Cycle Quality Improvement Project

**DOI:** 10.7759/cureus.99842

**Published:** 2025-12-22

**Authors:** Hamza Ahmed, Farah Mazhar, Zaynah Yousaf, Usman Akram, Sana Mazhar, Marium Rizwan

**Affiliations:** 1 Trauma and Orthopaedics, Salford Royal NHS Foundation Trust, Manchester , GBR; 2 General Practice, Salford Royal NHS Foundation Trust, Manchester, GBR; 3 Neurosurgery, Salford Royal NHS Foundation Trust, Manchester, GBR; 4 Haematology and Oncology, Calderdale and Huddersfield NHS Foundation Trust, Huddersfield, GBR; 5 Trauma and Orthopaedics, Salford Royal NHS Foundation Trust, Manchester, GBR

**Keywords:** discharge summary, documentation, follow-up, improving documentation, patient care, quality improvement

## Abstract

Introduction: Discharge summaries are critical for continuity of care, yet they often lack key information. Inadequate discharge documentation can lead to adverse events, medication errors, and poor follow-up of pending issues. We aimed to improve the completeness of discharge summaries-particularly follow-up instructions and communication to primary care-in a major trauma center, using standards from the Royal College of Physicians (RCP) Health Informatics Unit 2019 guidance.

Methods: We conducted a two-cycle quality improvement (QI) project in an orthopedic trauma unit of a Level I trauma center. In Cycle 1 (June-July 2025), we audited 33 discharge summaries against RCP-recommended content criteria (e.g. documentation of diagnoses, inpatient management, investigation results, medication changes, follow-up plans, general practitioner (GP) action items, and patient advice). Interventions implemented in August-September 2025 included staff education via posters, interactive teaching sessions, and team presentations to raise awareness of discharge summary standards. Cycle 2 (October 2025) reviewed 21 discharge summaries post-intervention. Following that, we compared documentation rates pre vs post intervention and used Fisher’s exact test for categorical improvements and Mann-Whitney U for ordinal scores.

Results: Follow-up documentation improved from 15.6% to 90.0% (absolute difference +74.4%, 95% confidence interval (CI) +58% to +91%, p < 0.0001). Complete documentation of new medication indications increased from 9.4% to 50.0% (absolute difference +40.6%, 95% CI +17% to +61%, p = 0.004). Comprehensive patient advice increased from 21.2% to 76.2% (+55.0%, 95% CI +31% to +79%, p = 0.0002).

Conclusion: Targeted educational interventions led to substantial improvements in discharge summary quality, particularly in follow-up instructions, medication documentation, and patient communication. Ensuring these elements are clearly documented helps bridge the transition from hospital to community care. Our project demonstrates that even in a busy trauma setting, focused efforts can align practice with recommended standards, improving handover to primary care and potentially patient outcomes. Sustained education and periodic audits are recommended to maintain high standards of discharge documentation.

## Introduction

Effective discharge summaries are essential for safe patient transitions from hospital to home or rehabilitation. These documents serve as the primary handover to general practitioners (GPs) and other community care providers, summarizing the hospitalization and outlining plans for follow-up [1]. High-quality discharge communication has been linked to reduced adverse events and medication errors post-discharge [2], as well as lower readmission rates and improved patient understanding of the care plan. [3]. However, studies have found that discharge summaries often omit critical information (e.g. pending investigation results, medication changes, follow-up tasks), leading to gaps in continuity of care [4,5].

Recognizing these issues, professional bodies have published standards and toolkits to improve discharge summary quality [1,6]. In 2019, the Royal College of Physicians (RCP) released guidance specifying core components for electronic discharge summaries (diagnoses, treatments, key investigation results, medication changes with reasons, follow-up arrangements, tasks for primary care, etc.). Despite such guidelines, compliance in practice is inconsistent as highlighted in one audit where only 55% of summaries met recommended content criteria. Time pressures and lack of training are major contributors. In one survey, 44% of hospital physicians admitted being too busy to prepare high-quality discharge summaries [7].

At our institution, discharge summaries for orthopedic trauma patients were noted to be inconsistently complete, especially regarding follow-up plans and communication to GPs. This posed a risk that important post-discharge tasks (e.g. GP removal of sutures, monitoring pending results) might be missed, and that patients could be unclear about their own care plans.

Aim

We set out to improve the completeness and quality of discharge summaries in our trauma unit, focusing on follow-up instructions and communication to primary care. The goal was to ensure each discharge summary contained all essential information per RCP guidelines, including a clear summary of the hospitalization, documentation of any medication changes (with reasons), explicit follow-up plans, and patient-friendly advice. We aimed to achieve measurable improvements over two audit cycles, hypothesizing that educational interventions could significantly increase compliance with recommended discharge summary content.

## Materials and methods

Setting

This project was conducted in the orthopedic trauma ward of a large tertiary care trauma center in the UK. Junior doctors primarily prepare discharge summaries using the electronic health record (EHR). The project was registered as a quality improvement (QI) initiative with the hospital’s clinical governance department, and formal ethical approval was not required.

Sampling method

A consecutive sampling method was used. Every discharge summary generated for eligible patients during the audit periods was included.

Timeline

The audit cycle durations (June-July 2025 for Cycle 1 and October 2025 for Cycle 2) were chosen based on feasibility and staffing considerations. These periods aligned with stable rota patterns for junior doctors and avoided peak winter pressures, ensuring consistent case-mix and allowing timely implementation of the educational intervention between cycles. As is typical in QI methodology, convenience sampling was used; however, the sample sizes reflected all eligible discharges produced during the audit windows and were adequate to identify significant changes in documentation practice. These time frames were, therefore, appropriate for detecting meaningful trends while remaining feasible within clinical workload constraints.

Inclusion criteria

Adults (≥16 years) admitted under the orthopedic trauma service, having an inpatient stay >24 hours, and being discharged directly to home, community hospital, or rehabilitation were included in this study.

Exclusion criteria

People who underwent elective orthopedic procedures, day‑case trauma patients, internal transfers to other inpatient teams, and patients whose discharges were generated by non‑orthopedic teams were excluded from this study.

Baseline audit

33 discharge summaries (June-July 2025) were reviewed against RCP-recommended content criteria covering key elements (clinical summary, investigations and pending results, medication changes, follow-up plans including any GP tasks, and patient advice/safety-netting). Each element’s completeness was rated on a 0-3 scale when applicable, defined as: 0 (Not mentioned); 1 (Mentioned but incomplete or unclear); 2 (Complete but lacking recommended detail); and 3 (Fully detailed, explicit, and aligned with RCP standards).

The 0-3 completeness scoring tool was developed locally based on the RCP 2019 discharge summary standards. Although not externally validated, it was internally reviewed by senior clinicians for face validity. All summaries were independently assessed by two reviewers (a senior trainee and a clinical fellow).

We also tracked seven-day and 30-day readmissions as balancing measures.

Interventions

In August-September 2025, we introduced multifaceted interventions to improve discharge summaries: an educational poster (a checklist of required components per RCP guidelines; see Appendices 1, 2), interactive teaching sessions (sharing baseline audit results and best-practice examples), routine reminders and feedback (a discharge summary checklist and one-on-one feedback for junior doctors after Cycle 1), and a campaign engaging the entire team to prioritize complete discharge documentation.

Follow-up audit**

In October 2025, we reviewed 21 discharge summaries (Cycle 2) post intervention using the same criteria to measure improvements.

Data analysis

We compared pre- vs post-intervention data using Fisher’s exact test for categorical variables and Mann-Whitney U for ordinal scores (p<0.05 considered significant).

## Results

Some discharge summary elements (e.g., documentation of outstanding investigations, GP action items, medication changes) were assessed only when clinically applicable. Therefore, denominators vary where patients did not have pending tests or medication changes. Relevance was determined based on review of the medical record and confirmation that the element applied to the patient’s admission.

Table 1 highlights the discharge summary content and documentation rates at baseline and after the intervention. 

**Table 1:** Discharge summary content documentation rates (baseline vs post intervention) Denominators vary because certain items were assessed only when clinically relevant (e.g., medication changes assessed only in patients with ≥1 change; outstanding tests assessed only when pending results existed). *: Item applicable only when relevant to the patient’s case. CI: Confidence interval; NS: Not significant; GP:

Content Element	Baseline Jun–Jul 2025	Post Intervention Oct 2025	P‑Value	95% CI for % Difference
Summary of health issues documented (Yes)	87.9% (29/33)	95.2% (20/21)	0.40 (NS)	−11% to +26%
Investigations & results documented (Yes)	66.7% (22/33)	90.0% (18/20)	0.10 (NS)	−4% to +49%
Outstanding tests mentioned *	44.4% (4/9)	75.0% (3/4)	0.56 (NS)	−38% to +84%
Inpatient management summary adequate (≥ Score 2)	72.7% (24/33)	95.2% (20/21)	0.03	+4% to +41%
Inpatient management summary fully detailed (Score 3)	30.3% (10/33)	76.2% (16/21)	0.001	+22% to +68%
Follow‑up plan fully detailed (Score 3) *	15.6% (5/32)	90.0% (18/20)	<0.0001	+58% to +91%
GP action instructions fully clear (Score 3) *	44.4% (4/9)	85.7% (6/7)	0.15 (NS)	−11% to +78%
Patient advice in lay terms (≥ Score 2)	75.8% (25/33)	81.0% (17/21)	0.76 (NS)	−22% to +32%
Patient advice in lay terms fully detailed (Score 3)	21.2% (7/33)	76.2% (16/21)	0.0002	+31% to +79%
Safety‑net advice included (Yes)	96.9% (32/33)	76.2% (16/21)	0.02	−39% to −2%
Safety‑net advice excellent (Score 3) *	46.9% (15/32)	93.8% (15/16)	0.001	+23% to +68%

After the intervention, nearly all discharge summaries contained a clearly documented summary of the patient’s issues and hospital course (95.2%). Documentation of pending investigations and follow-up tasks improved in frequency (absolute numbers were small for pending tests). The most dramatic improvement is seen in the quality of follow-up documentation (fully detailed plans rose from 15.6% to 90.0%, p<0.0001). Likewise, communication to patients (lay advice) saw a significant enhancement in completeness (p=0.0002). Although the percentage of summaries containing any safety-net advice decreased (97% to 76%, p=0.02), when present the advice was much more comprehensive (94% rated excellent vs 47% at baseline, p=0.001).

Table 2 highlights the documentation of medication changes and interventions. 

**Table 2:** Documentation of medication changes and interventions Denominators vary because certain items were assessed only when clinically relevant (e.g., medication changes assessed only in patients with ≥1 change; outstanding tests assessed only when pending results existed). *: Item applicable only when the patient had ≥1 medication change CI: Confidence interval; NS: Not significant

Medication Documentation Element	Baseline Cycle 1	Post‑Intervention Cycle 2	P‑Value	95% CI for % Difference
New medications started – fully documented (indication stated)	9.4% (3/32)	50.0% (9/18)	0.004	+17% to +61%
Medications stopped – fully documented (name + reason) *	33.3% (1/3)	100% (3/3)	0.20 (NS)	−9% to +133%
Medication dose changes – fully documented *	0% (0/4)	100% (1/1)	0.25 (NS)	−17% to +142%
Any medication change fully documented (corrected denominator)	8.6% (3/33)	52.4% (11/21)	0.001	+22% to +63%
7‑day readmission rate	6.1% (2/33)	4.8% (1/21)	1.00 (NS)	−16% to +14%
30‑day readmission rate	0% (0/33)	0% (0/21)	–	–

After the intervention, documentation of new medications improved significantly: half of new medications had their purpose documented, compared to <10% before (p = 0.004). Documentation for stopped and adjusted medications also improved to 100% in the few applicable cases (baseline numbers were small). The composite measure indicates that over half of patients in Cycle 2 had all their inpatient medication changes documented completely, versus <10% at baseline (p = 0.001). This suggests a major cultural shift in how diligently clinicians document medication information for handover. We did not observe any change in early re-admissions (both cycles had low rates).

Overall, the results demonstrate that our multifaceted intervention was successful in improving adherence to recommended discharge summary standards. Clinicians became far more diligent in including crucial follow-up information and explanations for care decisions, aligning practice closer with the ideal set forth by RCP guidelines.

Summary of findings

Marked improvement was seen in documentation of follow‑up plans, patient advice, and medication changes. Confidence intervals (CIs) support the robustness of key improvements.

## Discussion

In this quality-improvement project, we achieved marked enhancements in the content and clarity of discharge summaries in a major trauma setting. Following a targeted educational intervention, there were significant improvements in documenting follow-up plans, medication changes, and patient-focused advice. These findings underscore that relatively simple, low-cost interventions, like focused training and feedback, can substantially improve documentation quality and, by extension, continuity of care.

This project was designed and reported in alignment with SQUIRE 2.0 QI reporting guidelines to enhance methodological transparency and reproducibility.

Significance of improvements

The most consequential change was in follow-up documentation. Before the intervention, follow-up arrangements in discharge letters were often vague. After our interventions, virtually all patients had a clearly delineated follow-up plan in their summary, specifying the what, where, and when of next steps. This level of detail is crucial: clear follow-up instructions ensure patients receive appropriate ongoing care and that nothing “falls through the cracks” once they leave the hospital [8]. For example, if a trauma patient needs a repeat X-ray in two weeks to check fracture alignment, explicitly stating this in the discharge summary (and who is responsible for arranging/reviewing it) reduces the risk that the X-ray will be missed. Improved discharge communication has been shown to increase follow-up of pending tests and results [9].

Medication safety also benefited. Incomplete documentation of medication changes at discharge is a known contributor to post-hospital medication errors and adverse drug events [10]. By routinely including the indication for new medications and reasons for stopping medications, we made it easier for GPs to reconcile treatment plans. Primary care physicians want discharge summaries to clearly explain changes to regimens [11]. After our intervention, a GP reading the summary would readily understand why an antibiotic was discontinued or a beta-blocker dose was adjusted, without needing to retrieve hospital records. High-quality discharge summaries have been associated with fewer medication discrepancies and errors, so these improvements likely carry meaningful clinical benefits [12].

Comparison to other studies 

Prior discharge summary QI projects similarly achieved large improvements after introducing structured templates and feedback, reinforcing the effectiveness of these interventions. Our findings mirror other reports in the literature.

Importantly, we have demonstrated that even in a surgical/trauma ward, where discharge efforts often focus on wounds and follow-up appointments, it is possible to systematically improve the broader content of summaries (e.g. medication rationale, comorbidity documentation). Many published discharge summary improvements come from internal medicine settings [13]. Our work shows that surgical teams are equally capable of adopting better communication practices.

Intervention efficacy

We believe the combination of education and direct feedback was critical to our success.

Sustainability and next steps

We plan to integrate discharge summary best practices into the orientation for each new cohort of junior doctors, and to continue periodic audits to maintain high standards.

Limitations

This project had several limitations. It was a modest sample from a single unit, and we used a pre/post design without a control group, so there is potential for confounding factors or a Hawthorne effect influencing results. We did not directly measure patient or GP satisfaction with the discharge summaries (an area for future work), and the long-term sustainment of improvements is yet to be proven. Finally, while we tracked re-admissions, the study was not powered to detect changes in clinical outcomes, as many factors beyond documentation affect readmission risk. In addition to this, this project was conducted in a single orthopedic trauma unit within a tertiary center and used a specific EHR system. Documentation workflows, staffing structures, and EHR interfaces vary across hospitals and specialties; therefore, generalizability to other settings may be limited.

Implications for practice

Our project reinforces that improving discharge documentation is an achievable and vital component of delivering high-quality healthcare. Even small investments in clinician education and feedback can yield substantial improvements in the content of discharge summaries, which in turn may enhance patient safety and continuity of care.

## Conclusions

In summary, through a focused QI effort, we significantly improved the quality of discharge summaries in our trauma center, as evidenced by better documentation of clinical information, follow-up plans, and patient advice. By aligning practice with RCP’s recommended standards, our discharge summaries are now more reliable in communicating necessary information to both patients and primary care providers. We expect that these improvements will enhance patient safety and continuity of care, even if our short-term outcome metrics did not capture a difference in re-admissions. Future work will aim to maintain these gains and gather feedback from GPs and patients to further refine the process. Ultimately, our project adds to the evidence that improving discharge documentation is an achievable and important component of high-quality healthcare.

## Figures and Tables

**Figure 1:**
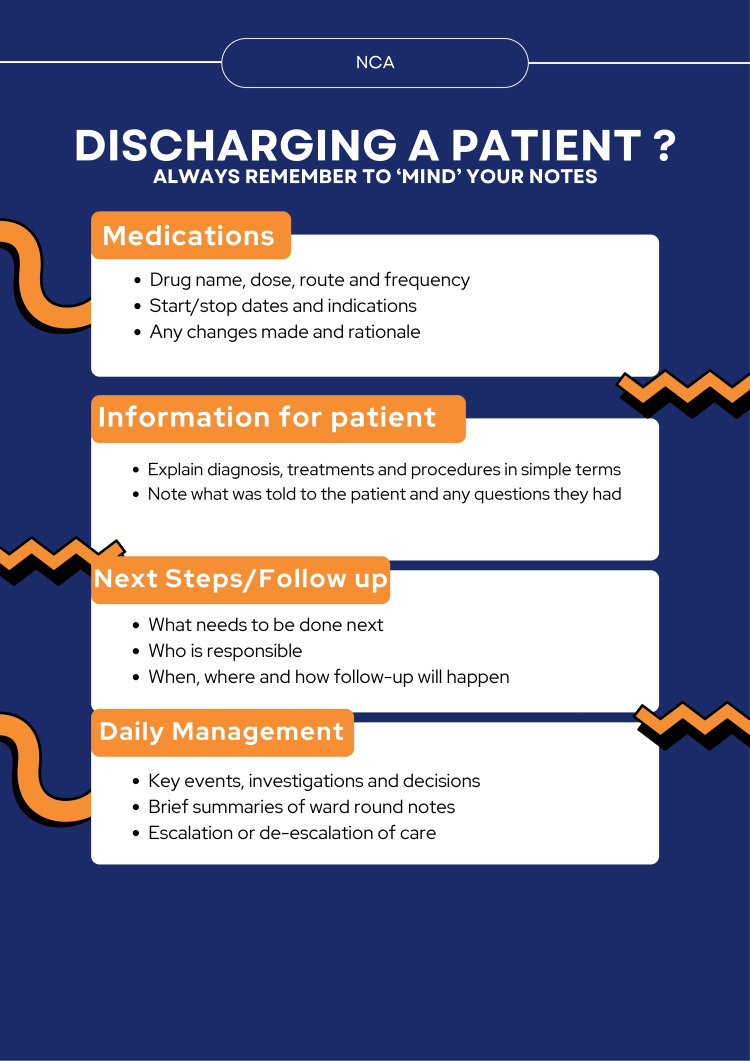
Poster used in intervention

**Figure 2:**
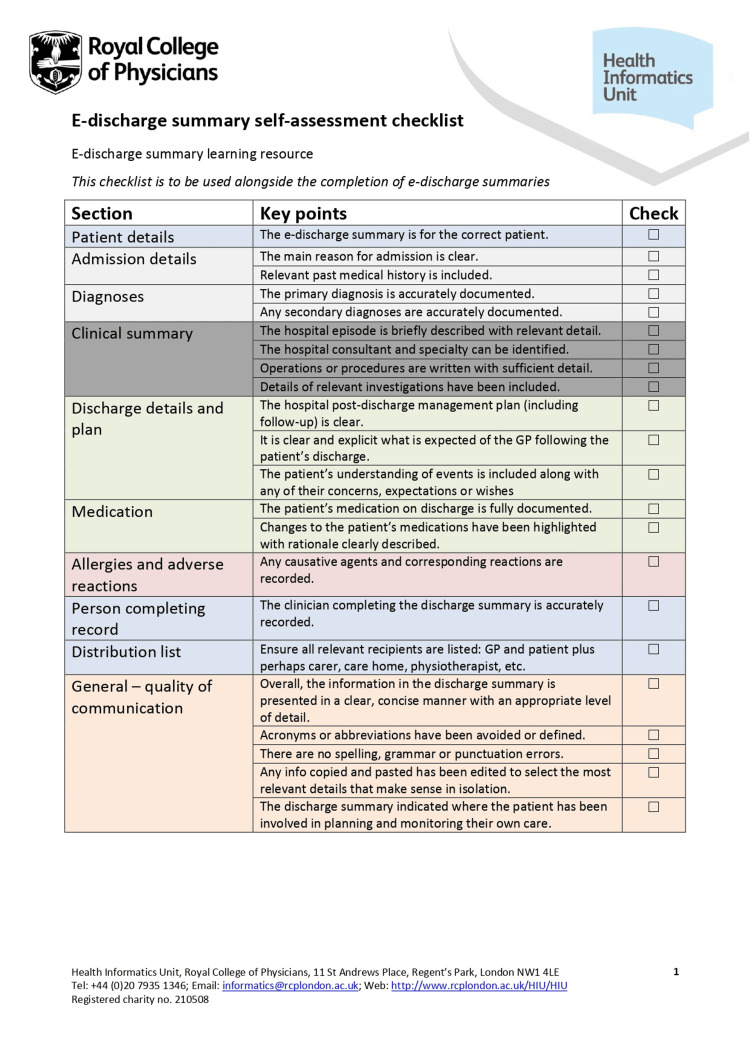
Discharge summary: self-assessment checklist

## Appendices

Appendix 1

Appendix 2
